# Migration and Health Policy: Applying the Nexus to Safety Issues of Migrants Crossing the Mediterranean Sea

**DOI:** 10.1002/puh2.70023

**Published:** 2024-12-31

**Authors:** Christopher Maatouk, Orestis Germanos, Yousef Khattab, Anna‐Maria Aad, Youssry Mohamed Elsawy Ibrahim Aboelhassan, Georges Gandour, Hamza Shafiq Hafeez, Paolo Miguel Manalang Vicerra, Shyam Sundar Budhathoki, David Lucas, Maria Luisa Canals, Don Eliseo Lucero‐Prisno

**Affiliations:** ^1^ Faculty of Medical Sciences Lebanese University Hadath Lebanon; ^2^ University of Cyprus Medical School Nicosia Cyprus; ^3^ Faculty of Pharmacy British University in Egypt Cairo Egypt; ^4^ Heliopolis University Cairo Egypt; ^5^ Faculty of Pharmacy Girne American University Girne Northern Cyprus; ^6^ Asian Demographic Research Institute Shanghai University Shanghai China; ^7^ School of Public Health Faculty of Medicine Imperial College London London UK; ^8^ ORPHY Laboratory Brest University Brest France; ^9^ Occupational and Environmental Diseases Center Teaching Hospital Brest France; ^10^ French Society of Maritime Medicine Brest Brest France; ^11^ University of Cadiz FUECA Cadiz Spain; ^12^ Sociedad Española de Medicina Marítima/Sanidad Marítima Tarragona Spain; ^13^ Department of Global Health and Development Faculty of Public Health and Policy London School of Hygiene and Tropical Medicine London UK; ^14^ Office for Research, Innovation and Extension Services Southern Leyte State University Sogod Southern Leyte Philippines; ^15^ Research and Innovation Office Biliran Province State University, Naval Biliran Philippines

**Keywords:** asylum seekers, global health, irregular migration

## Abstract

The Mediterranean Sea is the risky path utilized by migrants primarily seeking economic and physical security in Europe. Drowning is the most lethal among the many hardships they face on their way. In the pursuit of protecting individuals between countries of origin and destination, many European Union (EU) member states worked to decrease the number of migrants, most notably including the action involving the European agenda issued in 2015 when such migration peaked. Recognizing the nexus of migration and health policy underscores the imperative to develop comprehensive healthcare strategies that address the unique needs of migrant populations, promoting equitable access to healthcare services and safeguarding public health across borders. In an attempt to tackle the problem by its roots, European states established cooperation with third countries and provided multifaceted support, that is, economic assistance and personal safety, among others, to developing countries. They also relocated migrants to different parts of the region to decrease the stress faced by only selected countries. However, this plan, like other approaches, faced challenges. Despite their focus on enforcing the migration laws, the lack of unification of these laws hinders cooperation. Unifying the migration laws between EU members, a strict policy requiring the return of migrants at sea to their points of origin and making legal migration more accessible would render the process safer for all sides. A “New Pact on Migration and Asylum” has been proposed, but it still needs to be agreed on in full, and action must be taken. Moreover, these solutions could be joined by training programs in the countries of origin, in the hopes of securing employment in the country of destination, hence benefiting both countries. In addition, each EU country could partner with developing economies to create such job opportunities and build strong cooperative relations.

## 1 Introduction

Crossing the Mediterranean to Europe is the world's deadliest journey for migrants worldwide. It is responsible for the highest toll of casualties and missing individuals [1, 2]. It has caused at least 26,833 disappearances since 2014. [1] The peak death count was in 2016, with about 5136 lives lost. According to the International Organization for Migration (IOM), the first half of the year had a 58% increase in crossing attempts to Europe and twice as many deaths as in the previous year [3]. Most mortality cases occurred on the Central Mediterranean route (741 deaths), followed by the Western route (149 deaths) and then the Eastern route (6 deaths) (3). It is important to note that the actual figures may be higher as reports are based on the number of bodies found and survivors’ testimonies. There are also “invisible shipwrecks,” empty vessels being washed ashore [4].

The current article briefly describes the Mediterranean migration crisis along with recommendations concerning migrants’ health, security, and well‐being. The world's situation has been precarious in recent years, especially concerning migration, because the COVID‐19 pandemic has caused governments to become wary of cross‐border movements. The said crisis was related to events in 2014 when a large number of irregular migrants arrived in Europe [5]. The migration of Middle Eastern and sub‐Saharan African peoples to Europe at that point was not a novel event. Still, the volume of migrants became starker compared to previous years. Therefore, this article covers the period relating to the Mediterranean migration crisis involving economic migrants and refugees as they both use the same means to cross the sea. Reports and statistics published by respective countries’ agencies and international nongovernment organizations (NGOs) were utilized in order to ensure a more harmonized and reliable data environment.

## 2 Reasons Behind the Migration Through the Mediterranean Sea

Migration routes depend on proximity, country of destination, and cultural, historical, and linguistic linkage between the countries of origin and destination. It has been observed that sea travel may be a part of the migration process. Still, many migrants travel further and have different trajectories (6). Geographically, migrants take three routes: the Central Mediterranean route from North Africa to Italy and Malta, the Western Mediterranean route from Morocco to Spain, and the Eastern Mediterranean route from Turkey to Greece [6, 7]. Individuals have particular destinations apart from where their sea travel afforded them, and they stop and stay in certain countries because of poor health and the experience of crime, among other reasons.

Several factors push migrants to flee their countries of origin. An example of this is migrants from Tunisia, who were the most numerous as of January 2021 [7]. Considering its geographical location, migrants from Tunisia will take the Central Mediterranean route, where the destination is Italy. In 2017, there was a fourfold increase in Tunisians arriving in Italy [8]. This increase was due to the political instability and economic collapse that caused increasing inflation, high unemployment, and the selling of fisher folks’ boats to smugglers because of an invasive species of blue crabs hindering their livelihoods.

The forced movement of people has also been linked to climate conditions. Such was the case in Syria before the unrest occurred [9]. Consecutive dry winters were experienced in the country from 2006 to 2009, which happened again from 2010 to 2011 before the Syrian uprising. Food insecurity and economic hardship accelerated the displacement of rural agricultural families, which differed significantly from the usual rural‐to‐urban seasonal labor migration. Syrians were found to be among the majority to have crossed the Mediterranean Sea and attempted to find refuge in Europe [10].

The issues of the countries of origin were multifarious, and matters became confounded, ultimately forcing people to out‐migrate. Factors besides economic reasons push migrants to pursue employment opportunities and more secure living conditions abroad, especially after the Arab Spring and the Syrian civil war. Migrants also flee by sea because they need safety from physical and political threats, that is, war, human rights abuses, and persecution. Such conflicts in neighboring states forced 77% of migrants to Europe in general [6]. The said figure was exceptionally high in Greece, where many migrants were Syrians, such 91% cited that their movement was due to conflicts.

The demographic rise of Africa plays a significant role in driving emigration. Africa's population is projected to nearly double by 2050 [11], resulting in many young people entering the labor market. Limited economic opportunities and high unemployment rates in many African countries push individuals to seek better prospects elsewhere. The desire for improved economic and educational opportunities is a pull factor, attracting individuals to migrate to developed countries for a better life [12].

The drop in emigration costs due to increased access to information has also contributed to the recent rise in migration. Technological advancements, particularly the widespread use of the internet and social media, have made it easier for individuals to obtain information about migration opportunities. Potential migrants can now access information about job prospects, living conditions, and immigration procedures in destination countries. This increased information availability reduces the perceived barriers to migration, making it more attractive for individuals to undertake the journey.

The network effect of ethnic communities already settled in destination countries is another pull factor influencing migration decisions. Ethnic communities established in destination countries provide social networks, support systems, and cultural familiarity to newly arriving migrants. These networks play a crucial role in facilitating migration by providing information, assistance with settlement, and potential job opportunities [13]. The presence of established ethnic communities can make migration a more viable and desirable option for individuals seeking a sense of belonging and support in a new country.

## 3 Issues Faced by the Migrants

Migrants face issues even before leaving the shores of their countries of origin. Human rights abuse was prevalent in Libya, from arbitrary detentions, exploitation, and violence [14]. The said forms of violence force people to leave; however, if caught doing so, they are detained in horrific conditions [15]. These people often become exposed to ongoing conflict actions, that is, airstrikes for those in Tripoli. For those who can initiate their migration path, incidents that risk their health and safety were experienced. According to the Missing Migrants Project [1], about 93% of the total deaths since 2014 among migrants were caused by drowning, hazards during transit, and the harsh environment linked to lack of food and water, each accounting for about 1% of deaths, the remaining are from various reasons as violence, accidents, and lack of healthcare.

Drowning is a prevalent cause of death as overcrowding was often observed and reported among survivors, leading the vessels to capsize [1]. The boats are often quite simple in structure, not being made to withstand the sheer number of migrants. Other factors for unfortunate events compound the said type of transport being a risk. For one, it was frequent to leave during the night to avoid detection by coastguards. The weather was also not considered as long as the migrants had the opportunity to flee.

Journeys have also been noted to be lengthy, increasing people's exposure to any issue. Once migrants arrive at their countries of destination, they continue to be void of shelter and live in locations without proper sanitation or hygiene facilities [15]. Authorities in the respective countries were also reported to be hostile toward migrants, resulting in negative outcomes regarding their physical and mental health.

## 4 The Current State of the Migration Crisis

### 4.1 Reinforcing the Law

The European Union (EU) presented the 2015 European Agenda on Migration and the 2016 Migration Partnership Framework to create a common asylum policy between countries and to improve the path toward legal migration [16]. However, by 2021, such an asylum policy is still not standard at the EU level, and despite the European Commission releasing several legislative proposals to reform EU asylum policy, no text was adopted. Legal migration and asylum policies remained underdeveloped [16]. Other bodies that constitute the mechanism the EU has implemented were the European Asylum Support Office (EASO) and Frontex, among others. EASO was mandated to aid the process of asylum seekers, whereas the latter helps return irregular migrants.

Migrant smuggling has developed significantly since the migration crisis in 2015 (16). According to Europol and Interpol, over 90% of migrants paid smugglers to attempt to reach Europe [17]. This has made dismantling the smuggling business a core part of the EU's plan to limit migrants and one of the priorities for the period 2022–2025 of The European Multidisciplinary Platform Against Criminal Threats (EMPACT) [18]. In March 2020, the EU introduced military intervention through Operation Irini intending to gather information and patrol the Mediterranean area by planes. These actions were to disrupt the business model of human smuggling and trafficking networks.

The EU is also working closely with migrants’ countries of origin and transit, Libya in particular, to target smuggling networks [19]. As part of the cooperation with Libya, the EU provided the Libyan coast guards training, equipment, and support in 2016, intending to increase their ability to disrupt smuggling and trafficking in Libya and perform search and rescue (SAR) activities. According to the EU, the numbers of migrants intercepted by coast guards in 2017 and 2021 were 55,000 and about 26,300, respectively. As the figures show, the Central Mediterranean route has seen a decline in irregular arrivals from Libya between the said years [19].

Notable in the prior statement was the decline in the number of migrants who arrived in European jurisdiction through the Mediterranean migration routes. This may also have been due to the closing of borders due to the COVID‐19 pandemic since 2020 [20]. Crossing the sea and moving between transit hubs had become more restrictive due to cross‐border public health measures by the respective countries. Such scenarios resulted in dire situations for migrants as work opportunities were scarce, leading to greater financial insecurity. During the pandemic, basic survival provisions such as food, shelter, and hygiene facilities had also become insecure.

As mentioned before, cooperation with Libya had become a focal point of action for the migration crisis. However, an issue has been raised due to concerns regarding the treatment of migrants. It has been reported by the United Nations that certain entities, such as mercenaries and foreign fighters have targeted vulnerable groups. Different groups and migrants experienced enforced disappearances and various forms of hostilities and became especially vulnerable to patterns of violence, whether at sea or in detention centers. They had also become victims of trafficking. According to the IOM, some 10,000 people are currently detained in centers, with more than 1000 of these individuals being migrants requesting humanitarian aid [21]. There have been criticisms of the EU's policy of favoring externalization of protection obligations and calls to contend that the EU is abdicating its responsibility toward migrants to third countries [22, 23].

### 4.2 Search‐and‐Rescue Operations

In 2014, Italy ended Operation Mare Nostrum, which was part of the EU's humanitarian response at the onset of the migration crisis, whereby the operation included a SAR component with selected NGOs [24]. Italy was receiving all migrants from the SAR operations, which exacted a heavy toll since the burden was not shared by other EU member states [25]. Upon ceasing Operation Mare Nostrum, Italy then directed policies to be more restrictive measures against SAR NGOs (25).

After the cancellation of Operation Mare Nostrum, organizations, including Médecins Sans Frontières (MSF) and the Migrant Offshore Aid Station (MOAS), worked on save and rescue operations to augment the needs of migrants which was left by the Italian government [26]. They also maintained SAR through Frontex in order to address the shortcomings in humanitarian efforts for migrants through various operations like Operation Triton [24]. Problems persisted as Frontex performed a limited number of SAR activities. From 2016 to 2017, NGOs performed 26% of SARs, whereas the Italian Navy and Coast Guard did about 20% of rescues each, and ultimately, only 8% was performed by Frontex [16]. The latter organization's performance was attributed to its concentration of effort on border control and anti‐smuggling tasks (24).

### 4.3 Tackling the Issues in Countries of Origin and Destination

In order to tackle the economic incentive for migration, the EU drafted plans on partnering with third countries regarding technical, financial, and economic cooperation [16]. The actions that followed this general plan were to encourage people to stay in their home countries instead of choosing perilous migration routes. Various cooperation efforts to develop the skills of the people as well as the security of the country were among the actions being undertaken.

On the other hand, Italy and Greece were highly affected as they were the common destinations for Mediterranean migrants [16]. Hence, the 2015 agenda includes relocating migrants from these two countries to other member states or third‐country partners. An example of the latter is when Syrian migrants in Turkey in need of protection due to the war were resettled in EU member states in a voluntary humanitarian admission manner [16]. However, this plan would be opposed later on by countries such as Hungary, Romania, Slovakia, and the Czech Republic and would be scrapped by 2020, but not before successfully resettling some migrants to volunteering countries.

## 5 New Migration Pact 2020

### 5.1 Asylum Seekers

To address these pressing issues, the European Commission introduced the New Pact on Migration and Asylum in 2020 [27]. This pact hopes to tackle many of the problems posed by the migrant crisis.

The New Pact on Migration and Asylum proposes better screening procedures at the borders to enhance the identification of individuals needing international protection. This measure aims to ensure that asylum‐seekers receive adequate support while preventing the exploitation of migration channels by individuals without legitimate claims. Improved screening can help separate those in need of protection from economic migrants, facilitating a more efficient and fair asylum process.

The New Pact also emphasizes the importance of enhanced solidarity mechanisms, such as relocation or return sponsorship, to alleviate the pressure on member states heavily affected by migration flows. By distributing responsibilities more equitably, these mechanisms promote burden‐sharing and prevent overburdened countries from shouldering disproportionate responsibilities. Such solidarity measures can contribute to more effective and humane asylum systems across the EU.

Furthermore, the New Pact highlights the need for enhanced return coordination. This involves streamlining the return process for migrants who do not qualify for international protection, ensuring that returns are carried out humanely and with respect for human rights. Effective return coordination can contribute to maintaining the integrity of the asylum system and discouraging irregular migration while safeguarding the rights and dignity of migrants.

The further development of the asylum‐seekers database, Euordac, is proposed in the New Pact to improve information sharing and coordination among EU countries. This database would facilitate the exchange of asylum‐related data, including fingerprints and asylum decisions, leading to more efficient and secure asylum procedures. Euordac aims to enhance collaboration and promote more effective management of asylum processes within the EU.

### 5.2 Enforcing the Fight Against Migrant Smuggling

To combat migrant smuggling, the New Pact on Migration and Asylum calls for increased resources for EU SAR missions. Allocating more resources to these missions enhances the capacity to respond to distress situations, reduces the loss of life at sea, and ensures the safety of migrants during rescue operations. Strengthening SAR capabilities is crucial for safeguarding the lives and well‐being of migrants in perilous situations.

Additionally, the New Pact introduces a new EU Action plan against migrant smuggling. This action plan focuses on improving intelligence sharing, dismantling criminal networks, and enhancing cooperation among member states, neighboring countries, and international organizations. By enhancing coordination and joint efforts, this action plan aims to disrupt and deter migrant smuggling activities, thereby safeguarding the rights and safety of migrants.

### 5.3 Increasing Cooperation With Third Countries

Recognizing the need for international cooperation, the New Pact emphasizes strengthening partnerships with third countries. This approach seeks to address the root causes of migration, promote safe and orderly migration, and enhance migration management. The EU aims to establish comprehensive migration partnerships with countries of origin, transit, and destination through dialogue and tailored support.

Cooperation with third countries includes initiatives to address migration drivers, such as poverty, conflict, and climate change. The EU seeks to address the underlying factors that compel individuals to embark on dangerous migration journeys by investing in development, humanitarian aid, and long‐term solutions. Comprehensive cooperation with third countries can contribute to a more sustainable and holistic approach to managing migration.

### 5.4 Attracting Skills and Talents to the EU

To address economic development and demographic challenges, the New Pact proposes measures to attract highly skilled migrants to the EU. The Talent Partnership initiative aims to facilitate more effortless mobility for highly educated immigrants from selected countries, including the Western Balkans and Africa. By removing barriers to mobility and offering favorable conditions, the EU seeks to attract and retain talented individuals who can contribute to member states’ economic growth and innovation.

Completing the EU Blue Card Directive is another critical aspect of the New Pact. This directive streamlines the procedures for issuing work permits to highly skilled non‐EU nationals, making it easier for them to work and reside in the EU. The EU Blue Card aims to attract and retain professionals in high‐demand sectors, promoting knowledge transfer and expertise while addressing labor market needs.

Furthermore, the revision of long‐term residency permits, as proposed in the New Pact, would grant individuals once allowed the freedom of mobility within the Schengen area. This provision facilitates the integration of highly skilled migrants by allowing them to move freely across participating EU countries, promoting cross‐border collaboration and opportunities for personal and professional growth.

## 6 Recommendations

### 6.1 Assistance to Migrants in Distress

Migrants need protection regardless of being at sea or on land. The health of these individuals, particularly women and children, has to be secured because of the physical and mental strain brought about by traversing the Mediterranean Sea. Reports have shown people experiencing sunburn, skin diseases, various infections, and dehydration [28]. To address this, SAR missions need health specialists to attend to the migrants’ needs immediately. Medical doctors and midwives, among other health professionals, would assist the people. Psychiatric support will also be beneficial as migrants may have faced trauma from their point of origin and then face the difficulties of escaping and crossing the sea. Upon arrival on shore, adequate coverage of their health needs must also be ascertained. Such humanitarian assistance is beneficial during and after the COVID‐19 pandemic because of its transmissibility and the high risk of infection migrants experience.

### 6.2 Entry to European States

The New Migration Pact is an excellent first step in tackling the issue at hand. Although some agreements have been made to reduce unsafe migration, they still need to pass in full in order to see the results. There needs to be a standard asylum policy applied in all EU member states. Setting a list of rules among all countries would make cooperation easier as the plan would be well‐guided. Nevertheless, these standards must be accepted by all member states. This way, each member who agreed to the policy could be held accountable for not abiding by it.

The pact also proposes sending back migrants reaching the EU if they are ineligible on the basis of their background [29]. One way of doing this might include SAR missions with the plan of saving migrants in danger and returning them to their points of origin. NGOs might be able to help, potentially under some supervision, in order for it to follow procedures. This strict approach would deter individuals from taking that path. However, the asylum process should be done as fast as possible with the help of the different EU member states to the countries most afflicted with the migration issue.

In conjunction with the prior solution, creating new legal ways to migrate to the EU proposed by the New Pact on Migration and Asylum would push migrants to halt their maritime plans and opt for the safest and guaranteed procedure to reach their destinations. Centers placed in countries of origin ought to be aware of the reasons behind the migration and the desires of countries willing to receive qualified migrants.

### 6.3 Creating Economic Opportunities in the Countries of Origin

Countries with the highest numbers of migrants leaving ought to take them back. In return, the countries of destination would provide work permits to European labor markets. Thus, such a process would benefit the EU countries by sending back migrants and offering much‐needed employment opportunities and economic development. A possible idea to sustain that development is for each country or group of countries would be responsible for partnering with a specific country in need. The link could improve the partnership between the countries. Moreover, an EU state might allow the migrants who have become skilled in a particular job to continue in their own country. Hence, individuals of the third country would be incentivized to participate in the workplace, and countries receiving migrants would do so on a meritocracy basis and benefit from it. This would work perfectly for the new pact's revised Long‐Term Residence Directive and benefit all EU members.

## 7 Conclusion

The Mediterranean Sea migration crisis has resulted in high mortality and endangerment to individual safety. Since 2015, the number of migrants has declined tremendously, indicating improvements in handling the crisis. Although the aforementioned decline in trends in in‐migration to Europe has occurred, improving conditions in third countries may not be the reason. The COVID‐19 pandemic has shown how protective public health measures were utilized to protect the local populations in respective countries and aggravated the conditions experienced by irregular migrants along their transit or at the destination countries themselves.

Many lives are still at risk because of the situation in the countries of origin. Therefore, countries in the EU should agree on a plan that acts as a standard to deal with the asylum policy. This approach would make cooperation among EU member states and also with third‐country partners. Developing the capacity of third countries will be advantageous in terms of people's well‐being and societal advancement. The issue of the Mediterranean migration is as long‐lasting as the worldwide economic inequality, but with lives at risk, governments should take further responsibility to find a solution that helps all sides.

## Author Contributions

**Christopher Maatouk**: formal analysis, funding acquisition, investigation, methodology, writing–original draft, writing–review and editing. **Orestis Germanos**: formal analysis, funding acquisition, investigation, methodology, project administration, writing–original draft, writing–review and editing. **Yousef Khattab**: writing–original draft, writing–review and editing. **Anna‐Maria Aad**: writing–original draft, writing–review and editing. **Youssry Mohamed Elsawy Ibrahim Aboelhassan**: writing–original draft, writing–review and editing. **Georges Gandour**: writing–review and editing. **Hamza Shafiq Hafeez**: writing–review and editing. **Paolo Miguel Manalang Vicerra**: writing–review and editing. **Shyam Sundar Budhathoki**: CRediT contribution not specified. **David Lucas**: CRediT contribution not specified. **Maria Luisa Canals**: writing–review and editing. **Don Eliseo Lucero‐Prisno III**: conceptualization, supervision, writing–review and editing.

## Ethics Statement

The information we gathered did not need any ethics approval.

## Conflicts of Interest

The authors declare no conflicts of interest.

## Data Availability

Data sharing not applicable to this article as no datasets were generated or analyzed during the current study.
